# Circulating Exosomal miRNAs as Novel Biomarkers for Stable Coronary Artery Disease

**DOI:** 10.1155/2020/3593962

**Published:** 2020-12-11

**Authors:** Ping Zhang, Tao Liang, Yao Chen, Xuan Wang, Tianlong Wu, Zhixin Xie, Jianfang Luo, Yanhong Yu, Huimin Yu

**Affiliations:** ^1^The Second School of Clinical Medicine, Southern Medical University, Guangzhou, Guangdong 510515, China; ^2^Cardiovascular Department, Guangdong Cardiovascular Institute, Guangdong Provincial People's Hospital, Guangdong Academy of Medical Science, Guangzhou, Guangdong 510080, China; ^3^Guangdong Provincial People's Hospital, Guangdong Academy of Medical Sciences, School of Medicine, South China University of Technology, Guangzhou, Guangdong 510080, China; ^4^Key Laboratory of Regenerative Medicine of Ministry of Education, Department of Developmental and Regenerative Biology, College of Life Science and Technology, Jinan University, Guangzhou Guangdong 510632, China

## Abstract

Exosomal miRNAs are currently being explored as a novel class of biomarkers in cardiovascular diseases. However, few reports have focused on the value of circulating exosomal miRNAs as biomarkers for stable coronary artery disease (SCAD). Here, we aimed to investigate whether miRNAs involved in cardiovascular diseases in circulating exosomes could serve as novel diagnostic biomarkers for SCAD. Firstly, the serum exosomes were isolated and purified by the ExoQuick reagent and identified by transmission electron microscopy, western blot, and nanoparticle tracking analysis. Then, the purified exosomes were quantified by measuring the exosome protein concentration and calculating the total protein amount. Next, eight miRNAs involved in cardiovascular diseases, miR-192-5p, miR-148b-3p, miR-125a-3p, miR-942-5p, miR-149-5p, miR-32-5p, miR-144-3p, and miR-142-5p, were quantified in circulating exosomes from the control group (*n* = 20) and the SCAD group (*n* = 20) by quantitative real-time polymerase chain reaction (qPCR). Finally, the gene targets of the differentially expressed miRNAs were predicted, and the functions and signaling pathways of these targets were analyzed using an online database. The isolated exosomes had a bilayer membrane with a diameter of about 100 nm and expressed exosomal markers including CD63, Tsg101, and Flotillin but negatively expressed Calnexin. Both the exosome protein concentration and total protein amount exhibited no significant differences between the two groups. The qPCR assay demonstrated that among the eight miRNAs, the expression levels of miR-942-5p, miR-149-5p, and miR-32-5p in the serum exosomes from the SCAD group were significantly higher than that from the control group. And the three miRNAs for SCAD diagnosis exhibited AUC values of 0.693, 0.702, and 0.691, respectively. GO categories and signaling pathways analysis showed that some of the predictive targets of these miRNAs were involved in the pathophysiology processes of SCAD. In conclusion, our findings suggest that serum exosomal miR-942-5p, miR-149-5p, and miR-32-5p may serve as potential diagnostic biomarkers for SCAD.

## 1. Introduction

Coronary artery disease (CAD) is a heart disease caused by myocardial ischemia or hypoxia because of stenosis or occlusion due to coronary atherosclerosis. Although remarkable achievements have been made in drug and interventional therapies in recent decades, the prevalence and mortality of CAD are still on the rise in many countries [1, 2]. Nowadays, coronary angiography is the gold standard for the diagnosis of CAD in clinical practice, but it is expensive and invasive. Moreover, percutaneous coronary intervention (PCI) has become the main treatment of CAD, but evidence showed that about 3.4% to 12% of CAD patients after PCI would develop in-stent restenosis, which was a limitation for the long-term outcome [3, 4]. Based on different clinical characteristics and therapeutic principles, CAD can be classified into two categories that are stable CAD (SCAD) and acute coronary syndrome (ACS). ACS is characterized by angina that lasts more than 30 minutes and cannot be released spontaneously, accompanied by a significant change in electrocardiogram (ECG) and a sharp increase in the myocardial enzyme (such as troponin). Therefore, ACS can be easily diagnosed by combining the clinical symptoms and the results of ECG and myocardial enzyme. Unlikely, SCAD is sometimes uneasy to be diagnosed because SCAD presents as exertional angina and transient ECG changes that are difficult to be timely recorded. If not timely and effectively controlled, SCAD will progress to ACS, which is the leading cause of cardiovascular death worldwide [5]. Accordingly, it is urgent to seek for novel and specific biomarkers for the early diagnosis and effective treatment of SCAD.

MicroRNAs (miRNAs) are endogenous, single-stranded, and small noncoding RNAs that regulate gene expression at the posttranscriptional level [6]. Previous studies have demonstrated that some miRNAs are dysregulated in CAD and play key roles in the occurrence and development of CAD. For instance, circulating miR-206, miR-574-5p, and miR-33 expression was upregulated [7, 8], while miR-221, miR-130a, miR-155, and miR-26a-5p expression was downregulated in CAD patients [9, 10]. Among these dysregulated miRNAs, miR-574-5p, miR-155, and miR-26a-5p have been found to be involved in the proliferation and apoptosis of endothelial cells (ECs) and vascular smooth muscle cells (VSMCs) [10–12]. What is more, many miRNAs have been reported to have regulatory roles in the development of atherosclerosis [13, 14], the pathological basis of CAD. Hence, miRNAs have potential as diagnostic biomarkers and therapeutic targets for CAD.

Exosomes, a kind of endogenous extracellular vesicle with a diameter ranging from 40 to 100 nanometers (nm), contain different bioactive molecules such as RNAs and proteins and play an important role in intercellular communication [15, 16]. miRNAs are one of the most concerned molecules carried in exosomes. Recently, exosomal miRNAs have been widely explored as a novel class of biomarkers for various diseases including cardiovascular diseases. For example, circulating exosomal miR-103a-3p, miR-107, miR-320d, miR-486-5p, and let-7b-5p were identified to serve as biomarkers for atrial fibrillation (AF) progression [17]. The urinary exosomal miR-146a level was reported to be a good predictor of early renal injury in hypertension [18]. Exosomal miR-1915-3p, miR-4507, and miR-3656 expression levels had a high sensitivity to diagnose myocardial infarction (AMI) [19]. The decreased expression of miR-425 and miR-744 in the exosomes from heart failure patients and in the exosomes derived from fibrotic cardiac fibroblasts induced by Ang II, suggesting that the exosomal miR-425 and miR-744 have the potential to predict cardiac fibrosis and heart failure [20].

However, few reports have focused on the value of circulating exosomal miRNAs as biomarkers for SCAD. In this study, eight miRNAs, miR-192-5p [21], miR-148b-3p [22], miR-125a-3p [23], miR-942-5p [24], miR-149-5p [25], miR-32-5p [26], miR-144-3p [27], and miR-142-5p [28], which have been reported to be involved in the development of cardiovascular diseases were selected. The expression levels of these miRNAs in the circulating exosomes from the control and SCAD groups were detected by qPCR. Moreover, we used bioinformatics analysis to predict the gene targets of the differentially expressed miRNAs and analyze the functions and signaling pathways of these targets. The aim of this study was to investigate whether these miRNAs in circulating exosomes have potential as novel diagnostic biomarkers for SCAD.

## 2. Methods

### 2.1. Study Subjects

Study subjects were selected from the patients who underwent coronary angiography (CAG) for the evaluation of suspected CAD in the cardiology department of Guangdong Provincial People's Hospital. The inclusion criteria included 40-70 years old, gender unlimited, and diagnosis of SCAD or exclusion of CAD. Those with normal coronary angiograms were included in the control group, and those diagnosed with SCAD were included in the SCAD group. The diagnostic criteria for SCAD included [29]: (1) stenosis ≥ 50% in the left main coronary artery and ≥ 70% in one or more major coronary arteries (2) chest discomfort lasts no longer than 10 min. The exclusion criteria for all participants were as follows: cardiomyopathy, abnormal hepatic or kidney function (transaminase and creatinine levels exceeding the upper limit of normal reference value), malignancy diseases, autoimmune diseases, acute infection, surgery or trauma in the past half year, and patients who refused to participate in the study.

### 2.2. Sample Collection and Serum Isolation

Two milliliters (mL) of peripheral blood samples were obtained from each participant at about 6 a.m. (after an overnight fast) the day after admission. All the blood samples were collected in serum separator tubes containing separation gel and separated into serum fractions by centrifugation at 1000 g for 15 min after standing for half an hour. Serum specimens were frozen at -80°C until used.

### 2.3. Exosome Isolation and Purification

The serum samples were thawed at 4°C and then used to isolate exosomes with ExoQuick® ULTRA EV Isolation Kit (SBI, USA) according to the manual's instruction. Briefly, the serum samples were centrifuged at 3000 g for 15 min and then centrifuged at 12000 g for 10 min to remove cellular debris. All the centrifugation was performed at 4°C. Subsequently, 67 *μ*L of ExoQuick was added to 250 *μ*L of the centrifuged serum and incubated at 4°C for 30 min. The pellets of exosomes were collected by centrifuging the ExoQuick/serum mixture at 3000 g for 10 min. After that, the isolated exosomes were purified by elution of purification columns. If the purified exosomes were not immediately used for experiments, they should be stored at -80°C and avoid repeated freezing and thawing.

### 2.4. Transmission Electron Microscope

The morphology of the isolated exosomes was observed by transmission electron microscopy (TEM). Briefly, 5 *μ*L of exosome suspension was loaded onto a formvar-coated copper grid and fixed at least 5 min. Then, the exosomes were negatively stained with 2% uranyl acetate for 5 min. After being air-dried, the grids were visualized with TEM (JEM-1400 plus, JOEL, Japan) at 80 kV.

### 2.5. Western Blot

The isolated exosomes were lysed with RIPA lysis buffer (Dingguo, China), and the protein concentrations were measured by the BCA method. The proteins (10 *μ*g) were subjected to SDS-PAGE on a 12% polyacrylamide gel and transferred onto nitrocellulose membranes (Merck Millipore). After being blocked with 5% bovine serum albumin (BSA) for 1.5 h at room temperature, the membrane was incubated with anti-CD63 (1:200; sc-5275, Santa Cruz, USA), anti-Tsg101 (1:1000; YT4760, Immunoway, USA), anti-Flotillin (1:500; 15571-1, Proteintech, USA) antibody, and anti-Calnexin (1:1000, 2679s, CST, USA) overnight at 4°C. Then, the membranes were incubated with HRP-conjugated anti-rabbit IgG or HRP-conjugated anti-mouse IgG (1:2000; Sigma, Germany) for 1 h at 37°C. Finally, the protein bands were visualized by chemiluminescence using the ECL kit (Millipore) and quantified by the ImageJ software.

### 2.6. Nanoparticle Tracking Analysis

The size distribution and concentration of the isolated exosomes was analyzed by ZetaView (Particle Metrix, Germany). The sample chamber was cleaned with particle-free distilled water. Then the exosome samples were diluted 10000× in sterilized PBS. Subsequently, the diluted exosomes were slowly injected into the chamber and quantified using the nanoparticle tracking analysis software.

### 2.7. Determination of the Exosome Protein Concentration

The protein concentration of the isolated exosomes was determined with the BCA protein assay kit (Pierce, Thermo Fisher Scientific, USA). Briefly, the working reagent was prepared by mixing reagent A and reagent B at a ratio of 50 : 1 and added to a 96-well plate. The standard samples with a work range from 20 *μ*g/mL to 2000 *μ*g/mL were prepared by diluting albumin with PBS. The purified exosome samples lysed with RIPA lysis buffer were used as unknown samples. The standard and unknown samples were pipetted into the working reagent in the 96-well plate. Then, the 96-well plate was incubated at 37°C for 30 min, and the absorbance at 562 nm was measured on a microplate reader (Thermo, USA). The standard curve was constructed by plotting the absorbance for each albumin standard on *y*-axis against its concentration on the *x*-axis. Finally, the protein concentrations of the purified exosome samples were calculated according to the standard curve.

### 2.8. Real-Time Quantitative PCR Experiments

Total RNA from the serum exosomes was isolated by using the miRNeasy Serum/Plasma kit (Qiagen, Germany) according to the manufacturers' protocol. To normalize RNA content, 1 pmol of exogenous cel-miR-39-3p (RiboBio, China) was added into per 100 *μ*g exosomes diluted in the QIAzol lysis reagent. The expression levels of the eight selected miRNAs were detected by using miDETECT A Track™ miRNA qRT-PCR Starter Kit (RiboBio, China) with Bio-Rad CFX96 Real-Time System. The cycling parameters of the qPCR program were as follows: 95°C for 10 min (enzyme activation), followed by 45 cycles of 95°C for 2 s (denaturation), 60°C for 20 s (annealing), and 70°C for 10 s (extension). Primer sequences used for qPCR amplification of all the miRNAs were purchased from RiboBio. The miRNAs expression levels were normalized to cel-miR-39-3p and expressed as log_10_ (2^−[CT (miR)–CT (cel-miR−39-3p)]^). For all qPCR experiments, the samples were run in quadruplicate.

### 2.9. Bioinformatics Analysis

The prediction of miRNA target genes was performed by an online bioinformatics tool, miRwalk (http://mirwalk.umm.uni-heidelberg.de/). The filtering conditions of target genes are set by default. We also used this tool for functional annotation and signal pathway analysis of target genes.

### 2.10. Statistical Analysis

Statistical analysis was performed using SPSS 24.0 statistical software (Chicago, IL, USA), and the graphs were generated using GraphPad Prism 7.0. The normal variables were presented as mean ± SD and compared with the two-tailed Student *t* tests, and nonnormal variables were presented as median with interquartile range and compared with nonparametric test. In addition, categorical variables were assessed by chi-square tests. Moreover, receiver operating characteristic (ROC) curves were constructed for the differentially expressed miRNAs in the diagnosis of SCAD. *P* < 0.05 was considered to be statistically significant.

## 3. Results

### 3.1. Identification of the Isolated Exosomes from Serum

**Figure 1:**
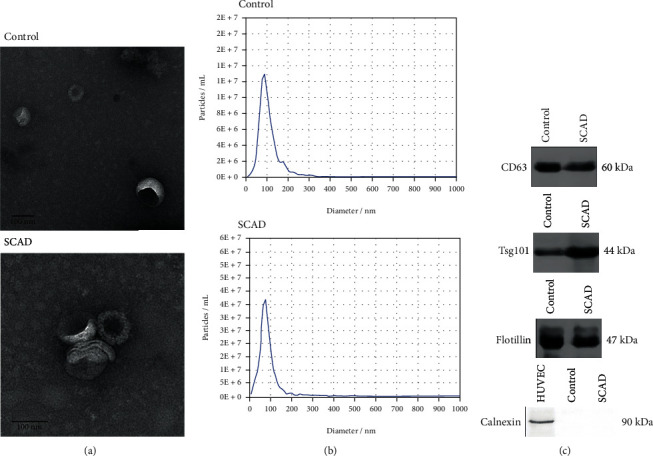
Identification of the exosomes isolated from the serum of the control subjects and the SCAD patients. (a) Transmission electron microscopy showing the morphology of exosomes. (b) Nanoparticle tracking analysis showing the concentration and size of the isolated vehicles. (c) Western blot analysis showing the expression of positive and negative markers including CD63, Tsg101, Flotillin, and Calnexin in the isolated vehicles. SCAD: stable coronary artery disease.

Exosomes isolated from the serum of the control and SCAD groups were identified by TEM, western blot, and nanoparticle tracking analysis. TEM imagines showed that isolated exosomes had a bilayer membrane with a diameter of about 100 nm (Figure 1(a)). Nanoparticle tracking analysis indicated that over 90% of the isolated vesicles were within 100 nm in diameter (Figure 1(b)). Western blot analysis revealed that the isolated vesicles positively expressed exosomal marker proteins including CD63, Tsg101, and Flotillin but negatively expressed Calnexin (Figure 1(c)), and there were no significant differences in the expression of CD63, Tsg101, and Flotillin of the exosomes from the control group and SCAD group (Supplementary Figure 1). These results confirmed that most of the isolated vesicles are exosomes and could be used for the following experiments.

### 3.2. Clinical Characteristics of the Study Population

The clinical characteristics of the study population including 20 control subjects and 20 SCAD patients are shown in Table 1, and pharmacological treatments of the participants are shown in Supplementary Table 1. The SCAD patients had significantly higher hs-cTnT levels than control subjects, while no significant differences were observed in other indexes between the control subjects and SCAD patients.

### 3.3. The Quantification of the Exosome Protein

**Figure 2:**
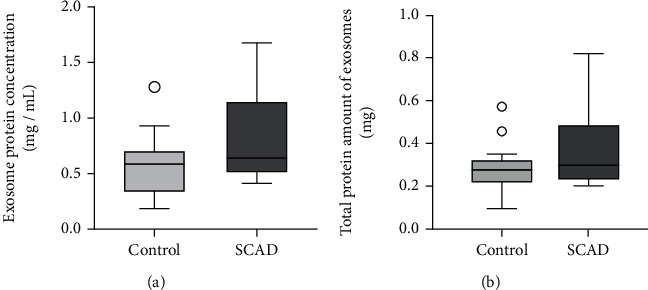
Box-and-whisker plots of the quantification of exosomes from the study subjects. (a) and (b) exhibit the protein concentration and total protein amount of the exosomes from the control group (*n* = 20) and SCAD group (*n* = 20), respectively. Data are presented as median with interquartile range and compared with nonparametric test. SCAD: stable coronary artery disease.

The exosome protein concentrations of the control and SCAD groups were measured by the BCA method, and the total protein amount was calculated by multiplying the protein concentration by the volume of the isolated exosomes. There were no significant differences in the protein concentration and total protein amount between the exosomes from the control and SCAD groups (control vs. SCAD: 0.58 (0.34, 0.68) vs. 0.64 (0.52, 1.15), *P* = 0.09, and 0.28 (0.22, 0.31) vs. 0.29 (0.24, 0.48), *P* = 0.16, respectively, Figure 2), indicating that the exosome levels in the serum were not different between the two groups.

### 3.4. qRT-PCR Identification of Exosomal miRNAs with Different Expression

**Figure 3:**
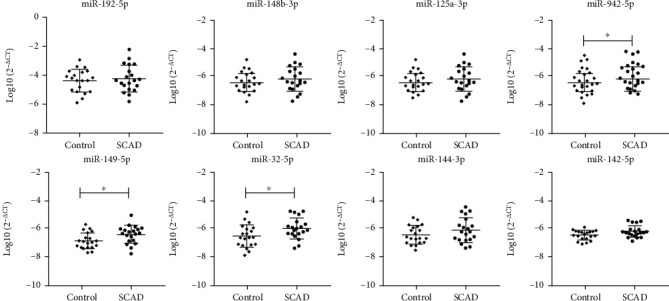
Comparisons of relative expression of the select exosomal miRNAs between exosomes from the control subjects (*n* = 20) and SCAD patients (*n* = 20). The expression levels of the select exosomal miRNAs were detected by qRT-PCR. Values were normalized to cel-miR-39-3p and were expressed as log10 (2[CT(miR)-CT(cel-miR-39-3p)]). Data are presented as the mean ± SD and compared by *t* test (^∗^*P* < 0.05). SCAD: stable coronary artery disease.

To identify the exosomal miRNAs that were differentially expressed between the control subjects and SCAD patients, the expression levels of the eight selected miRNAs in serum exosomes from the control group (*n* = 20) and SCAD group (*n* = 20) were analyzed by qRT-PCR. As shown in Figure 3, there are no significant differences in the expression levels of miR-192-5p, miR-148b-3p, miR-125a-3p, miR-144-3p, and miR-142-5p between the two groups. However, the expression levels of miR-942-5p (control vs. SCAD: −6.2 ± 0.9 vs. −5.6 ± 0.8, *P* = 0.038), miR-149-5p (control vs. SCAD: −6.7 ± 0.7 vs. −6.1 ± 0.7, *P* = 0.021), and miR-32-5p (control vs. SCAD: −6.1 ± 0.9 vs. −5.4 ± 0.9, *P* = 0.032) were significantly increased in the SCAD group compared to the control group (Figure 3).

### 3.5. ROC Curve Analysis

**Figure 4:**
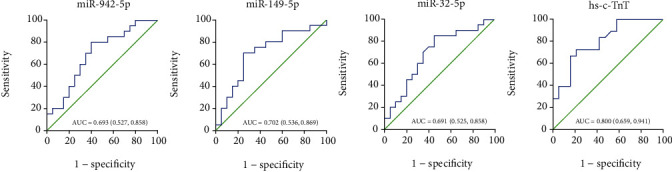
ROC curve analysis of the differentially expressed exosomal miRNAs and hs-cTnT between the control and SCAD groups. The 95% confidence interval of the AUC value was indicated in parentheses. SCAD: stable coronary artery disease; Hs-cTnT: high-sensitive (hs)-cardiac troponin T.

We further constructed ROC curves for the differentially expressed miRNAs and hs-cTnT to evaluate their diagnostic value for SCAD. miR-942-5p, miR-149-5p, miR-32-5p, and hs-cTnT for SCAD diagnosis exhibited AUC values of 0.693, 0.702, 0.691, and 0.800 (Figure 4 and Table 2), suggesting that they could be used as diagnostic biomarkers for SCAD.

### 3.6. Gene Ontology and Pathway Enrichment Analysis of the miRNA Targets

**Figure 5:**
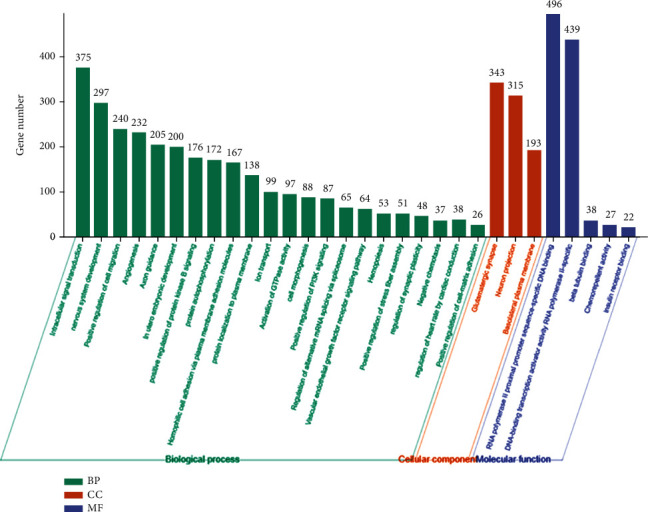
Top thirty significantly enriched GO categories for the differentially expressed miRNA targets. Bar charts showing the enrichment of differentially expressed miRNAs target genes in biological process, cellular component, and molecular function. *Y*-axis represents gene number, and *x*-axis represents the GO category. GO: Gene Ontology; BP: biology process; CC: cellular component; MF: molecular function.

**Figure 6:**
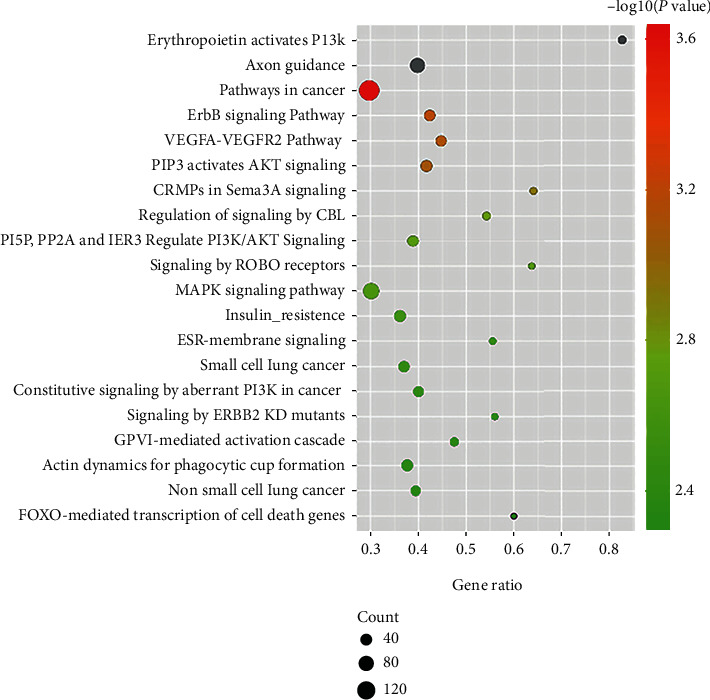
Top twenty significantly enriched signaling pathways for differentially expressed miRNA targets. Bubble chart showing the enrichment of differentially expressed miRNA target genes in signaling pathways. *Y*-axis represents the pathway, and *x*-axis represents gene ratio, which meant the ratio of the amount of target genes enriched in the pathway and the amount of all genes annotated in this pathway. The size and color of the dot represent the amount of target genes enriched in the pathway and enrichment significance, respectively.

The target genes of miR-942-5p, miR-149-5p, and miR-32-5p were predicted by using the miRWalk database. We found that miR-942-5p targets 2310 genes, miR-149-5p targets 2448 genes, and miR-32-5p targets 217 genes, and any two of these three miRNAs have some common target genes (Supplementary Figure 2). In order to investigate the potential function of the differentially expressed miRNAs, gene ontology (GO) and pathway enrichment analysis of their target genes were subsequently carried out. GO enrichment analysis indicated that the target genes functioned in intracellular signal transduction, nervous system development, positive regulation of cell migration, angiogenesis, and other GO categories (Figure 5). Reactome and Kyoto Encyclopedia of Genes and Genomes (KEGG) pathway enrichment analysis revealed that the target genes were significantly enriched in pathways in cancer, MAPK signaling pathway, axon guidance, and so forth (Figure 6). Several of these significantly enriched pathways were involved in the pathophysiology processes of CAD, such as VEGFA–VEGFR2, PI3K, and MAPK signaling pathway. These results revealed that miR-942-5p, miR-149-5p and miR-32-5p might involve in the pathogenesis of CAD.

## 4. Discussion

In the present study, we selected eight miRNAs that had been reported to be involved in cardiovascular and investigated whether these miRNAs in circulating exosomes had the potential to sever as biomarkers for SCAD. We found that three miRNAs (miR-942-5p, miR-149-5p, and miR-32-5p) involved in cardiovascular diseases were significantly upregulated in the serum exosomes from SCAD patients, and these exosomal miRNAs had good predictive value for SCAD diagnosis. Furthermore, GO and pathway enrichment analysis revealed that some of the predictive targets of these miRNAs were involved in the pathophysiology processes of SCAD. Our findings suggest that exosomal miR-942-5p, miR-149-5p and miR-32-5p may sever as novel diagnostic biomarkers for SCAD.

Increasing evidence suggests that the quantification of circulating exosomes could be used as a biomarker for some diseases [30, 31]. According to the guidelines, the quantification of exosomes can be achieved by the quantification of particle number, total protein amount, total lipids, or total RNA [32]. In our study, we quantified the isolated exosomes by measuring the exosome protein concentration and calculating the total protein amount. However, no significant differences were found in the exosome protein concentrations and total protein amount between the control and SCAD groups, indicating the exosome levels were not different between the two groups. Since the exosome protein concentrations varied greatly in the study population, the finding needs to be verified in studies with larger sample sizes.

For several advantages, circulating exosomal miRNAs have emerged as promising biomarkers for various diseases. Firstly, miRNAs are stable and detectable in the circulating exosomes. Circulating miRNAs packaged in exosomes can escape from nuclease degradation due to the protection of the lipid bilayer membrane of exosomes and remain in a highly stable state [33]. What is more, previous evidence has suggested that most of the miRNAs detectable in serum are enriched in exosomes [34]. Secondly, exosomal content (including miRNA and other molecules) varies according to the microenvironment of their parent cells [35], so the levels of exosomal miRNAs may reflect the pathophysiologic conditions of the body. Thirdly, clinical evidence suggested that the circulating exosomal miRNAs may be more sensitive in predicting diseases than circulating miRNAs. For example, a previous study showed that miR-208a expression levels were significantly increased in both exosomes and serum of ACS patients, but the sensitivity of miR-208a in serum for ACS diagnosis was inferior to that in exosomes [36]. Another study demonstrated that the expression of miR-146a in circulating exosomes but not in the plasma was significantly upregulated in patients with heart failure [37]. Therefore, circulating exosomal miRNAs are of great potential as biomarkers for diseases.

However, little was known about the potential of circulating exosomal miRNAs as biomarkers for CAD, especially SCAD. A previous study showed that the expression levels of exosomal miR-208a were increased in ACS patients and the high expression of miR-208a were associated with the decreased 1-year survival rate, suggesting that exosomal miR-208a could sever as biomarker for diagnosis and prognostic assessment of ACS [36]. Moreover, it was demonstrated that exosomal miR-1915-3p, miR-4507, and miR-3656 were decreased in the serum exosomes of AMI patients and these miRNAs were predictive for AMI [19]. In addition, a recent study found that serum exosomal miR-126 and miR-21 levels were increased in ACS and AMI patients and exosomal miR-126 had a strong correction with the severity of coronary artery stenosis [38]. The above studies suggest that the expression of circulating exosomal miRNAs is dysregulated in ACS and AMI. However, whether circulating exosomal miRNAs are differentially expressed in SCAD remains to be studied. In this study, we found that exosomal miR-942-5p, miR-149-5p, and miR-32-5p were significantly increased in SCAD patients and had good predictive value for SCAD diagnosis. Our findings add to the accumulating evidence that suggests that circulating exosomal miRNAs have potential as novel diagnostic biomarkers for CAD.

miRNAs have been recognized as important regulators in the development and progression of CAD [39, 40]. However, the roles of miR-942-5p, miR-149-5p, and miR-32-5p in the pathophysiology of CAD are rarely reported. Previous reports demonstrated that miR-149-5p could alleviate endothelial injury caused by oxidized low-density lipoprotein (ox-LDL) through inhibiting PAPP-A [41] and reduce endothelial dysfunction induced by high glucose through decreasing TNF-*α* and ER stress marker expression [42]. Moreover, it was found that miR-149-5p were involved in the regulation of efferocytosis in advanced atherosclerosis [25]. As for miR-32-5p, one report revealed that the serum miR-32-5p expression was increased in AMI patients, which might indicate myocardial damage, endothelial injury, and inflammatory activation [43]. Another report demonstrated that miR-32-5p was increased in CAD patients with coronary vascular calcification and it had a critical role in modulating vascular calcification progression. With respect to miR-942-5p, its relationship with CAD has not been reported in the literature yet. Because the role of these miRNAs in the pathogenesis of CAD was little known, they may be of great interest for future research.

Atherosclerosis, the most common pathological substrate of CAD, is a chronic progressive process characterized by lipid deposition in the arterial wall. The pathogenesis of atherosclerosis is complex, involving endothelial dysfunction, macrophage adhesion and migration, VSMC migration and proliferation, oxidative stress, and inflammation [44]. In the present study, we employed GO and pathway enrichment analysis to investigate whether miR-942-5p, miR-149-5p, and miR-32-5p have potential roles in the pathogenesis of CAD. We found that the miRNA targets were significantly enriched in various GO categories and signaling pathways. Among the top 30 most significant GO categories, the biological processes of cell migration, angiogenesis, and VEGFR signaling pathway (Figure 6) are common pathophysiological processes in the development of atherosclerosis [12, 45–47]. Moreover, some of the top 20 most significant pathways have been widely reported to be involved in the pathogenesis of atherosclerosis. For instance, VEGFA–VEGFR2 signaling pathway mainly plays a role in the regulation of angiogenesis [46]. Both PI3K signaling pathway and MAPK signaling pathway can generate diverse responses in different cell types in the development of atherosclerosis, including angiogenesis, proliferation, and migration of VSMCs and macrophages, inflammation, and oxidative stress [48–52]. Therefore, the results of the GO and pathway enrichment analysis revealed that miR-942-5p, miR-149-5p, and miR-32-5p might play important roles in the pathogenesis of CAD by regulating the abovementioned processes and signaling pathways, which could provide important clues for functional experiments on the molecular mechanism of SCAD in the future.

As far as we know, this study showed for the first time that circulating exosomal miRNAs had the potential as biomarkers for SCAD, which may provide an auxiliary tool for the diagnosis of SCAD and novel therapeutic targets for SCAD. Although some efforts were made in this study, several limitations should be taken into account. Firstly, in our study, three of the eight selected miRNAs were significantly upregulated in the circulating exosomes of SCAD patients, which suggested that our methods were effective. However, there might be other exosomal miRNAs with differential expression in SCAD patients, but these miRNAs were not included in our study. To identify as many differentially expressed exosomal miRNAs as possible in SCAD, it is advisable to explore the miRNA expression profile in exosomes from SCAD patients by using sequencing. Secondly, the serum exosomes were isolated and purified by using the ExoQuick reagent. Compared with ultracentrifugation, the ExoQuick reagent produced a higher yield but a lower purity of exosomes [53]. However, exosomes isolated by ExoQuick reagent were similar in quality to those isolated by the ultracentrifugation method [53]. Thirdly, the sample size in our present study is small, and our findings are warranted to be validated in another cohort. Fourthly, we preliminarily found that the exosomal miR-942-5p, miR-149-5p, and miR-32-5p might play crucial roles in the pathogenesis of CAD by bioinformatics analysis. Functional experiments are warranted to uncover the regulatory mechanism of these miRNAs in the development of CAD.

In general, our findings suggest that serum exosomal miRNAs such as miR-942-5p, miR-149-5p, and miR-32-5p may serve as novel diagnostic biomarkers for SCAD. Further studies are needed to reveal the function and mechanism of these exosomal miRNAs in the occurrence and development of SCAD.

## Figures and Tables

**Table 1:** Clinical characteristics of the study population.

Index	Control (*n* = 20)	SCAD (*n* = 20)	*P* value
Gender (male/female)	12/8	14/6	0.51^^^
Age (years)	57 (52, 62)	64 (52, 68)	0.10^#^
BMI (kg/m^2^)	24.0 ± 3.0	24.7 ± 2.9	0.44
Diabetes (%)	3 (15.0)	6 (30.0)	0.26^^^
Hypertension (%)	8 (40.0)	7 (35.0)	0.74^^^
SBP (mmHg)	137.7 ± 15.1	138.4 ± 15.8	0.89
DBP (mmHg)	83.1 ± 13.8	80.6 ± 8.4	0.49
Creatinine (*μ*mol/L)	64.9 ± 9.0	71.6 ± 14.6	0.09
Glucose (mmol/L)	5.0 (4.6, 5.3)	5.3 (4.7, 6.4)	0.17^#^
ALT (U/L)	20.0 (15.0, 27.0)	24.0 (14.0, 33.0)	0.45^#^
AST (U/L)	19.6 ± 5.0	21.4 ± 5.7	0.29
LDL-C (mmol/L)	3.0 ± 0.8	3.1 ± 0.9	0.56
HDL-C (mmol/L)	1.1 ± 0.3	1.0 ± 0.2	0.22
TG (mmol/L)	1.9 (1.4, 2.6)	1.8 (1.5, 2.0)	0.55^#^
TC (mmol/L)	4.9 (3.5, 5.5)	4.6 (4.1, 5.3)	1.00^#^
hs-cTnT (pg/mL)	5.4 (5.0, 7.1)	8.6 (6.4, 55.2)	<0.01^#^

SCAD: stable coronary artery disease; BMI: body mass index; SBP: systolic blood pressure; DBP: diastolic blood pressure; ALT: alanine aminotransferase; AST: aspartate aminotransferase; LDL-C: low-density lipoprotein cholesterol; HDL-C: high-density lipoprotein cholesterol; TG: triglyceride; TC: total cholesterol; hs-cTnT: high-sensitive- (hs-) cardiac troponin T; ^^^chi-square test; ^#^nonparametric test.

**Table 2:** AUC analysis of the differentially expressed miRNAs and hs-TnT between the control and SCAD groups.

miRNA	AUC	95% CI	Sensitivity (%)	Specificity (%)	*P* value
miR-942-5p	0.693	(0.527, 0.858)	80.0	60.0	0.037
miR-149-5p	0.702	(0.536, 0.869)	70.0	75.0	0.028
miR-32-5p	0.691	(0.525, 0.858)	85.0	55.0	0.039
hs-cTnT	0.800	(0.659, 0.941)	66.7	84.2	0.002

ROC: receiver-operating-characteristic; AUC: area under curve; CI: confidence interval; SCAD: stale coronary artery disease; hs-cTnT: high-sensitive (hs)-cardiac troponin T.

## Data Availability

All the data used to support the finding of the present study are presented in the article. The original data of the study are available from the corresponding author upon request.

## Supplementary Materials

Supplementary Materials Supplementary Table 1: Pharmacological treatments of the participants. Supplementary Figure 1: Quantification of exosomal markers in the isolated exosomes from the control and SCAD groups. Supplementary Figure 2: The regulatory network between the differentially expressed miRNAs and their target genes. The blue and orange roundness represented the differentially expressed miRNAs and their target genes, respectively. The target gene number was indicated in parentheses.
