# Supplementary material for: Circulating Exosomal miRNAs as Novel Biomarkers for Stable Coronary Artery Disease
Source: Biomed Res Int. 2020 Dec 11;2020:3593962. doi: 10.1155/2020/3593962 (PMC7748912; doi:10.1155/2020/3593962)

**Supplementary material**

**Supplementary Table 1**: Pharmacological treatments of the participants.

| **Medicine** | **Control (n=20)** | **SCAD (n=20)** |
|---|---|---|
| Aspirin | 0 | 3 |
| ACEI/ARB | 4 | 3 |
| Calcium Channel Blockers | 3 | 0 |
| Diuretics | 1 | 2 |
| Statins | 0 | 3 |
| Hypoglycemics | 3 | 6 |

ACEI: angiotensin-converting enzyme inhibitor; ARB: angiotensin receptor blocker.

**Supplementary Figure 1**: Quantification of exosomal markers in the isolated exosomes from the control and SCAD groups.

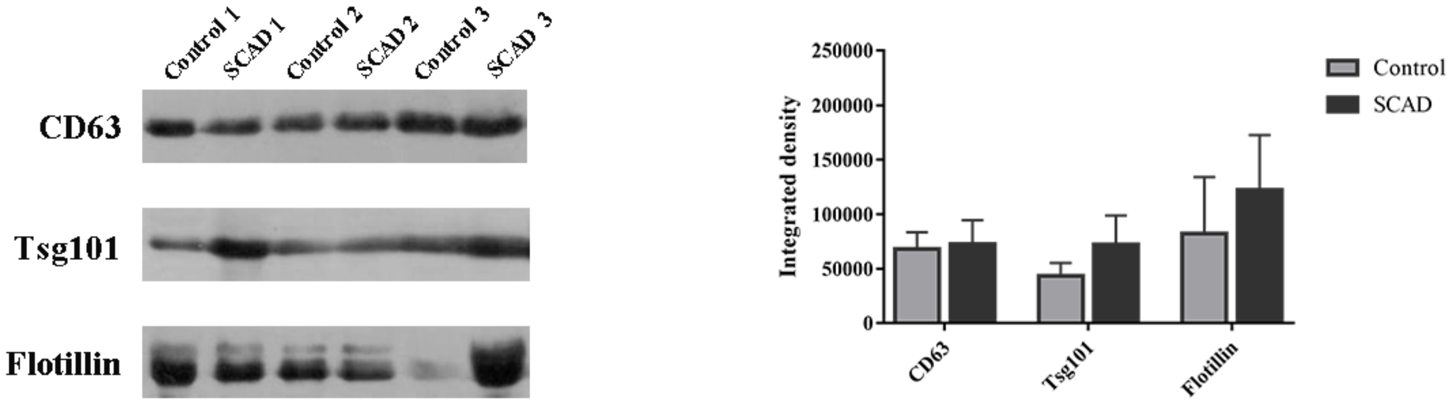

**Supplementary Figure 2: The regulatory network between the differentially expressed miRNAs and their target genes**. The blue and orange roundness represented the differentially expressed miRNAs and their target genes, respectively. The target gene number was indicated in parentheses.

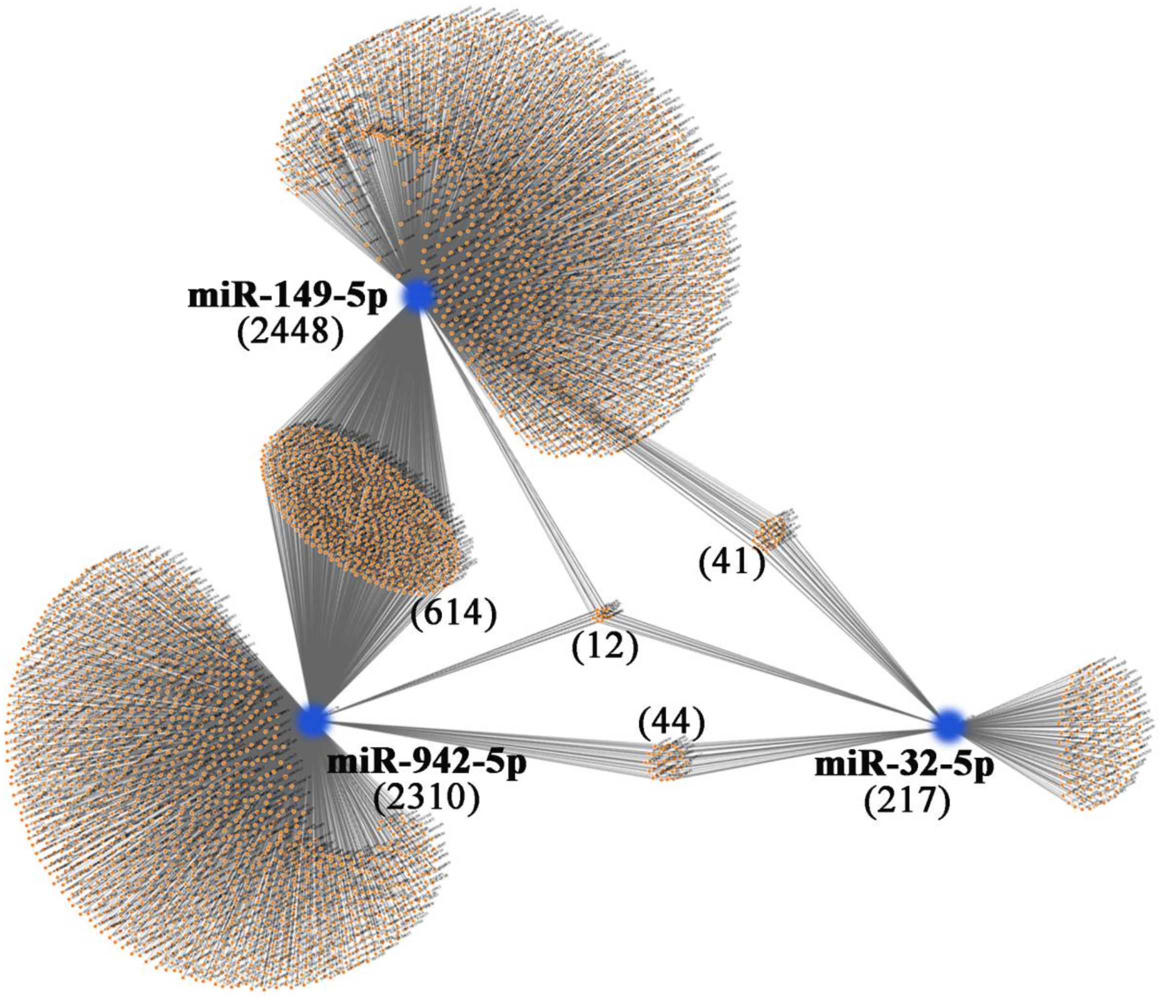

Supplement: Supplementary Materials — Supplementary Table 1: Pharmacological treatments of the participants. Supplementary Figure 1: Quantification of exosomal markers in the isolated exosomes from the control and SCAD groups. Supplementary Figure 2: The regulatory network between the differentially expressed miRNAs and their target genes. The blue and orange roundness represented the differentially expressed miRNAs and their target genes, respectively. The target gene number was indicated in parentheses. [file 3593962.f1.pdf]
